# Pelacarsen and lipoprotein(a) apheresis in secondary prevention: the Lp(a)FRONTIERS APHERESIS trial

**DOI:** 10.1093/eurheartj/ehag073

**Published:** 2026-02-21

**Authors:** Klaus G Parhofer, Ulrich Julius, Anna Laura Herzog, Thilo Krüger, Julia Weinmann-Menke, Gunnar H Heine, Hui Cao, Jessica Schorr, Philippe Ferber, Jing Wang, Enrico Cristante, Björn Müller-Edenborn, Anja Vogt, Volker Schettler, Elisabeth Steinhagen-Thiessen

**Affiliations:** Department of Medicine IV—Grosshadern, LMU University Hospital, LMU Munich, Munich, Germany; Department of Internal Medicine III, University Hospital Dresden at the Technische Universität Dresden, Dresden, Germany; Transplantation Center, Department of Nephrology, University Hospital Wuerzburg, Würzburg, Germany; DaVita Clinical Research Germany, Duesseldorf, Germany; Department of Nephrology and Clinical Immunology, Uniklinik of the RWTH Aachen, Aachen, Germany; Department of Internal Medicine I and Research Center of Immunotherapy, University Medical Center Mainz, Mainz, Germany; Medical Department II, AGAPLESION MARKUS KRANKENHAUS, Frankfurt, Germany; Novartis Pharmaceuticals Corp, East Hanover, NJ, USA; Novartis Pharma GmbH, Nuremberg, Germany; Novartis Pharma AG, Basel, Switzerland; Novartis Pharmaceuticals Corp, East Hanover, NJ, USA; Novartis Pharmaceuticals UK Ltd, London, UK; Novartis Pharma AG, Basel, Switzerland; Department of Medicine IV, LMU University Hospital, LMU Munich, Munich, Germany; Center of Nephrology Göttingen GbR, Göttingen, Germany; Department of Endocrinology and Lipid Clinic, Charite-Universitätsmedizin Berlin, Augustenburger Platz I, 13353 Berlin, Germany; Department of Clinical Chemistry, Lipid Clinic Universitätsmedizin Rostock, Ernst Heydemann Str. 5, 18057 Rostock, Germany; Friede Springer Cardiovascular Prevention Center, Charite-Universitätsmedizin Berlin, Hindenburgdamm 30, 12203 Berlin, Germany

**Keywords:** Lipoprotein apheresis (LA), Lipoprotein(a) [Lp(a)], Cardiovascular disease, Pelacarsen

## Abstract

**Background and Aims:**

Lipoprotein apheresis (LA) is the only approved treatment for patients with elevated lipoprotein(a) [Lp(a)]. The Lp(a)FRONTIERS APHERESIS trial investigated whether pelacarsen reduces the need for LA in patients from Germany with elevated Lp(a) and established cardiovascular disease (CVD).

**Methods:**

Adult patients with Lp(a) levels >60 mg/dl who had undergone ≥35 LA sessions in the prior year were randomized to receive pelacarsen 80 mg or placebo every 4 weeks for 52 weeks. Weekly LA sessions were performed if the Lp(a) measurement from the prior visit was >60 mg/dL. The primary endpoint was the rate of performed LA sessions normalized to the weekly LA schedule (the number of actual LA sessions divided by the number of planned LA sessions during the 52-week period). Secondary endpoints were time to LA avoidance (for ≥24 consecutive weeks) and total LA avoidance from week 12 to week 52.

**Results:**

Fifty-one patients were randomized (mean age 61.7 years, mean Lp(a) at baseline 85.4 mg/dL, and mean 44.0 LA sessions in the past 12 months), with 25 of 26 (96.2%) in the pelacarsen arm and 23 of 25 (92.0%) in the placebo arm completing the study. Baseline characteristics were generally balanced between treatment arms. Pelacarsen reduced the mean rates of LA (0.16 vs 0.93 in placebo, odds ratio 0.006, 95% confidence interval [CI] 0.003, 0.013; *P* < .0001) and substantially increased the hazard of achieving LA avoidance (hazard ratio: 88.3; *P* = .0014; median time to achieve LA avoidance: 6.1 weeks) and total LA avoidance (odds ratio: 163.2; *P* = .0005). The placebo-adjusted Lp(a) change from baseline at week 52 was *−*72% (95% CI: *−*79%, *−*61%; *P* < .0001). Treatment emergent adverse events were similar between arms, except for mostly mild injection site erythema (pelacarsen 38.5%; placebo 0%).

**Conclusions:**

Pelacarsen is a highly effective and well-tolerated Lp(a)-targeted therapy that substantially reduces the need for LA in patients with elevated Lp(a) and established CVD.

**ClinicalTrials.gov, identifier:**

NCT05305664.

## Structured Graphical Abstract

**Graphical Abstract:**
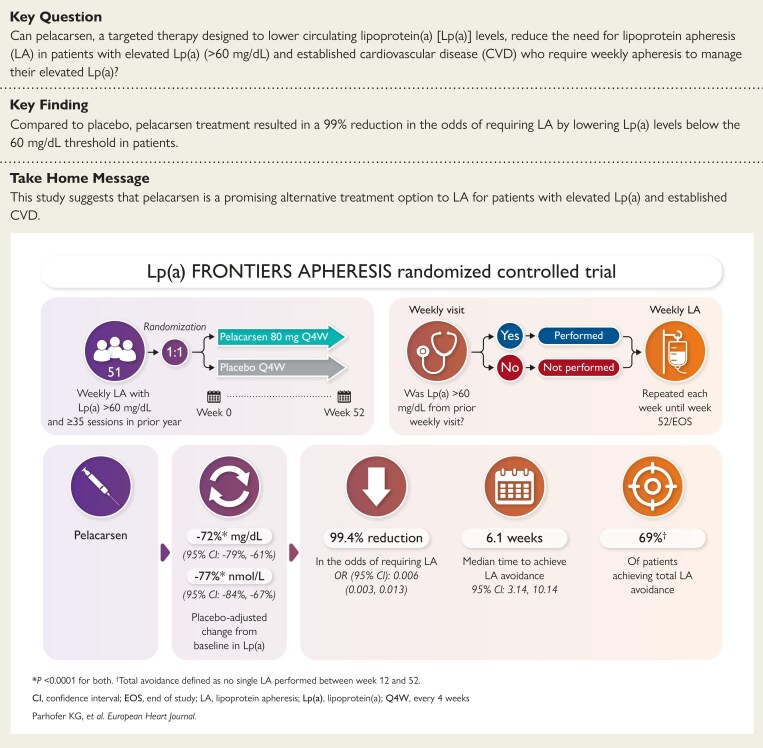
The Lp(a)FRONTIERS APHERESIS randomized controlled trial.

**See the editorial comment for this article ‘Lowering lipoprotein(a): inhibiting production or enhancing clearance?’, by M. Ammar and S. Mora, https://doi.org/10.1093/eurheartj/ehag132.**

## Introduction

Elevated lipoprotein(a) [Lp(a)] is a highly prevalent genetically driven dyslipidaemia present in approximately one in five individuals of the general population, and in one in four individuals with atherosclerotic cardiovascular disease (ASCVD).^1–3^ Epidemiological, genetic, and Mendelian randomization studies have established elevated Lp(a) as an inherited, independent, and causal risk factor for cardiovascular disease (CVD),^4–7^ with lifestyle measures such as diet and exercise having minimal influence on serum levels.^8,9^

Currently, there are no pharmacological therapies approved for lowering Lp(a). Lipoprotein apheresis (LA) is the only approved treatment to lower the Lp(a) burden in very high-risk patients with established CVD.^10,11^ Widespread use of LA is restricted to specialized centres in selected countries and further constrained by high costs and time commitments that require patients to adhere to an invasive and regularly scheduled treatment procedure.^10,12^ Germany is a leading country in the use of LA for the treatment of patients with elevated Lp(a), as the therapy is reimbursed for the secondary prevention of progressive CVD in patients with isolated elevated Lp(a) (defined as Lp(a) > 60 mg/dl [∼120 nmol/l] and normal low-density lipoprotein cholesterol [LDL-C] levels).^13^ In the initial phase following an LA session, Lp(a) levels are lowered by up to 75%. Depending on LA intervals, time-averaged reductions are less substantial and average to approximately 40% even with a once-weekly apheresis schedule.^12,14,15^

Pelacarsen, a hepatocyte-directed GalNAc-conjugated antisense oligonucleotide, selectively binds to apolipoprotein(a) [apo(a)] mRNA, preventing apo(a) protein synthesis and thereby inhibiting Lp(a) production. In a Phase 2b trial pelacarsen 20 mg weekly reduced Lp(a) levels by 80% in patients with elevated Lp(a) levels (≥60 mg/dl) and established CVD.^16^ The primary objective of this study was to evaluate the effect of pelacarsen 80 mg every 4 weeks vs placebo on the need for LA in patients with elevated Lp(a) and established CVD who were undergoing weekly LA in Germany.

## Methods

### Study design and participants

Lp(a)FRONTIERS APHERESIS (NCT05305664) was a randomized, placebo-controlled, double-blind, multicentre, Phase 3 trial across 13 hospital sites in Germany that enrolled patients from August 2022 to January 2024 and completed in January 2025.

Adults aged ≥18 to ≤80 years with established CVD, undergoing weekly LA for isolated elevated Lp(a) (defined as Lp(a) > 60 mg/dL and LDL-C in the normal range) and who had a minimum of 35 sessions within the past 52 weeks prior to randomization were included (see Supplementary data online, *Figure S1*). Patients were required to have a pre-apheresis Lp(a) level >60 mg/dl at screening visit week 2. Established CVD was defined as any of the following clinical manifestations of ASCVD: spontaneous prior myocardial infarction (MI), prior ischaemic stroke, clinically significant symptomatic peripheral artery disease (PAD) or clinically significant coronary artery disease (CAD). Patients with uncontrolled hypertension, active liver disease, planned cardiovascular procedures, heart failure (New York Heart Association class IV) or recent (˂12 weeks) proprotein convertase subtilisin kexin/type 9 (PCSK9) inhibitor use were excluded. A full list of eligibility criteria is provided in Supplementary data online, *Table S1*.

This trial was approved by an institutional review board and/or independent ethics committee, and was conducted according to the Declaration of Helsinki, and the International Conference on Harmonization Good Clinical Practice guidelines. All participants provided written informed consent prior to enrolment.

### Intervention

Following a 2-week screening period comprising two weekly LA sessions, eligible patients underwent an additional LA session and were randomized 1:1 (block size 4; no stratification) to receive pelacarsen 80 mg subcutaneously or placebo every 4 weeks during the double-blind treatment period of 52 weeks. Allocation concealment was ensured using an interactive response technology system for randomization, blinding of patients and all personnel involved in the study including study treatment that was identical in packaging, labelling, appearance etc. Lp(a) measurements after the randomization visit were reported to investigators semi-quantitatively (≤60 mg/dl or >60 mg/dl). LA was only performed at a visit if the pre-apheresis Lp(a) level from their prior visit was >60 mg/dl (see Supplementary data online, *Figure S1*). At screening, baseline, and weekly visits, samples for Lp(a) analysis were collected prior to, and after LA (if conducted); if the visit also included pelacarsen or placebo treatment, these were administered after LA. All participants, investigator staff, persons performing study assessments, and the clinical trial team remained blinded for the duration of the trial until database lock.

### Trial endpoints

The primary endpoint was the normalized rate of LA sessions performed over 52 weeks in the pelacarsen and placebo treatment arms. This normalized rate of LA sessions was calculated as the number of actual LA sessions received, divided by the number of planned LA sessions during the 52-week period, which is 52 for patients who completed all study visits, or pro-rated for those who discontinued early. This rate could range from 0 to 1, with 0 indicating that the patient had skipped all planned LA sessions, and 1 indicating that the patient had received all planned sessions. A rate of 0.8 indicated that the patient received 80% of planned LA sessions (and skipped 20% of planned sessions). The rate was derived with adjustments for imputed missing LA sessions (e.g. due to patient dropout).

Secondary endpoints included: (i) time to LA avoidance, where LA avoidance was defined as ≥24 consecutive weeks of no LA until end of study; (ii) total avoidance of LA from week 12 to week 52; and (iii) change from baseline to week 52 in log-transformed Lp(a) levels (measured prior to planned LA as measured in mg/dL). Information on other exploratory efficacy endpoints are publicly available on ClinicalTrials.gov NCT05305664.

### Efficacy and safety assessments

The decision as to whether LA should be performed during the weekly visit was exclusively determined by the Lp(a) mass-measurement from the prior visit. Lp(a) levels were measured in both mass units (mg/dl) and molar units (nmol/l) by a central laboratory (see Supplementary data online, Appendix  *S2*). Blood samples were also collected to assess changes in other lipid biomarkers and all results of Lp(a) and other lipids/apolipoproteins were blinded to patients, site staff and the study team. Unless stated otherwise, all samples at all timepoints were taken pre-apheresis. To mitigate a potential increase in LDL-C in patients achieving avoidance of apheresis secondary to Lp(a) values <60 mg/dl, LDL-C was measured monthly and reported to sites unblinded, with the option to adapt lipid-lowering therapies as needed.

Safety was assessed throughout the trial by continuously monitoring treatment emergent adverse events (TEAEs) during the on-treatment period, which is defined as the period from the date of first injection to 16 weeks after the last actual injection of study drug (16 weeks is equivalent to five half-lives for pelacarsen), the death of the participant, or the participants study end date, whichever comes first.

### Statistical analyses

The estimated sample size of 46 patients in a 1:1 randomization was considered for demonstrating the superiority of pelacarsen over placebo in reducing the normalized rate of LA sessions at a one-sided 2.5% alpha level (see Supplementary data online, Appendix  *S1*). The study would have >80% power based on an assumed normalized rate of LA sessions of 0.2 for the pelacarsen arm (which was based on data of the phase II study),^16^ and a plausible range of 0.67–0.75 for the placebo arm, while accounting for a 10% drop-out rate. The study eventually randomized 51 patients, with 26 patients in the pelacarsen arm, and 25 in the placebo arm. No interim analyses were conducted. The primary and secondary efficacy analyses were based on the full analysis set (FAS), defined as all randomized patients. All safety analyses were based on the safety set comprising of all randomized patients who received double-blind study treatment.

The normalized rate of LA sessions was analysed using a fractional logit model^17^ with the logit link under quasi-binomial distribution (to adjust for over-dispersion), including treatment as a factor and log-transformed baseline Lp(a) level as a covariate. The treatment difference was determined using the odds ratio and 95% confidence intervals (CI).

Time to LA avoidance, defined as time from randomization to the first visit among 24 consecutive weeks without apheresis for those achieving avoidance (‘event’), or the earliest of the week 28 visit date, death date, end of study date for those not achieving avoidance (‘censored’), was analysed using a Cox regression model with treatment as a factor and log-transformed baseline Lp(a) as a covariate. The 95% CI for the median time was based on the Kaplan–Meier estimator with the complementary log–log transformation. The total avoidance of LA from week 12 to week 52 was analysed using a logistic regression model with treatment as a factor and log-transformed baseline Lp(a) as a covariate. To address the complete separation scenario where there were no events in the placebo arm, Firth's penalized maximum likelihood approach was utilized to reduce bias and ensure the models for the above analyses produced finite, reliable estimates.^18^ Change from baseline to week 52 in log-transformed Lp(a) was analysed using an analysis of covariance (ANCOVA) model with treatment as a factor and log-transformed baseline Lp(a) as a covariate.

A hierarchical inferential approach was deployed to control for overall type I errors. As the primary hypothesis was rejected at the 2.5% alpha level, the three secondary hypotheses were tested using the Hommel testing method.^19^

Missing data were imputed to ensure robust and reliable analysis results (details given in Supplementary data online, Appendix  *S3*). All the *P*-values in this manuscript are one-sided unless specified otherwise. All analyses were performed with SAS version 9.4 (SAS Institute, Cary, NC, USA) or R version 4.3.1 (R Foundation for Statistical Computing, Vienna, Austria).

## Results

### Baseline demographic and clinical characteristics

**Figure 1:**
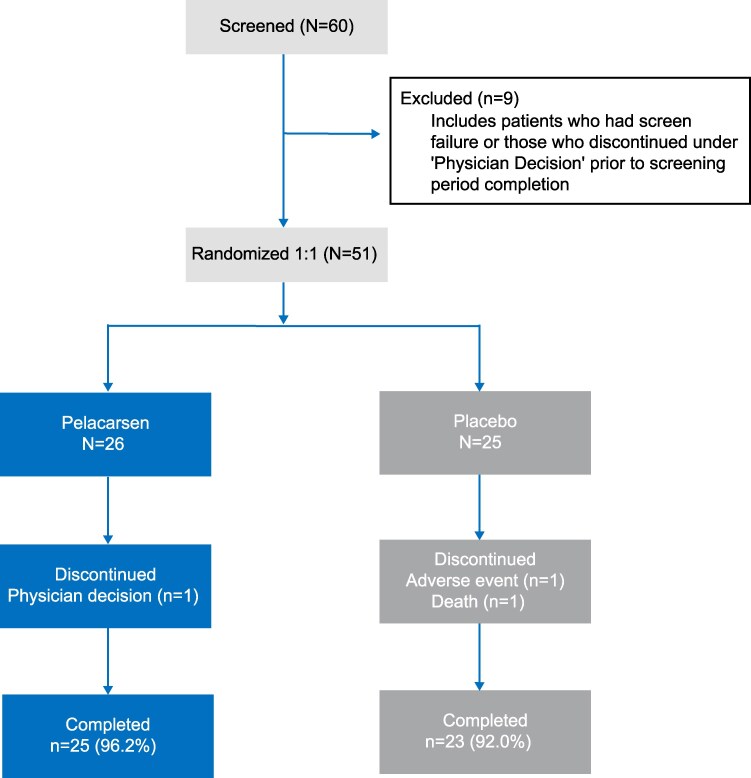
Patient disposition. The number of patients who were screened, randomized, allocated to treatment arms, discontinued, and completed the trial is shown. Percentages were calculated by *n*/*N* for each treatment arm. *n*/*N*, number of patients who completed the trial/number of patients randomized

A total of 60 patients were screened, of whom 51 were enrolled. Twenty-six were randomized to the pelacarsen arm and 25 to the placebo arm. A total of 25 patients (96.2%) from the pelacarsen arm and 23 patients (92.0%) from the placebo arm completed the double-blind period to week 52 (*Figure 1*). The mean (±SD) duration of exposure to study drug was 345.5 (±56.6) days in the pelacarsen arm and 337.9 (±77.1) days in the placebo arm, corresponding to a total duration of exposure of 24.5 and 23.1 patient-years, respectively (see Supplementary data online, *Table S2*).

Patient demographic and clinical characteristics are summarized in *Table 1*. The median (Q1, Q3) age of the total population was 62 (57, 66) years, of whom 39 patients (76.5%) were male. Among all 51 patients, the most common qualifying events were CAD in 47 patients (92.2%) and prior MI in 40 patients (78.4%). Additionally, 10 patients (19.6%) had PAD, and 6 patients (11.8%) had a history of ischaemic stroke. Overall, 45 patients (88.2%) experienced more than one qualifying cardiovascular event or condition. Background use of lipid-lowering therapy, and baseline measurements of lipids (total cholesterol, LDL-C, HDL-C, and triglycerides) and apoB were balanced between treatment arms. Median (Q1, Q3) baseline measurements of Lp(a) were numerically higher in the pelacarsen arm (90 mg/dl [64, 109]) compared to the placebo arm (74 mg/dl [66, 92]), with a similar trend observed when Lp(a) was measured in nmol/L.

**Table 1:** Baseline demographic and clinical characteristics

Characteristics	Pelacarsen *n* = 26	Placebo *n* = 25	Total *n* = 51
Age (years) [median (Q1, Q3)]	62 (57, 66)	63 (58, 65)	62 (57, 66)
Men, *n* (%)	20 (76.9)	19 (76.0)	39 (76.5)
White, *n* (%)	26 (100)	25 (100)	51 (100)
BMI (kg/m^2^) [median (Q1, Q3)]	27.9 (25.8, 30.4)	27.8 (23.4, 30.2)	27.8 (24.6, 30.4)
msSBP (mmHg) [median (Q1, Q3)]	130 (122, 156)	128 (118, 139)	129 (118, 142)
msDBP (mmHg) [median (Q1, Q3)]	74 (67, 87)	76 (72, 83)	75 (69, 85)
Sitting pulse rate (beats/min) [median (Q1, Q3)]	64 (61, 72)	67 (60, 74)	65 (60, 73)
Qualifying CVD event—*n* (%),			
Myocardial infarction	21 (80.8)	19 (76.0)	40 (78.4)
Ischaemic stroke	1 (3.8)	5 (20.0)	6 (11.8)
Peripheral artery disease	5 (19.2)	5 (20.0)	10 (19.6)
Coronary artery disease	25 (96.2)	22 (88.0)	47 (92.2)
>1 qualifying event^a^	24 (92.3)	21 (84.0)	45 (88.2)
MI and ischaemic stroke	1 (4.2)	3 (14.3)	4 (8.9)
MI and PAD	1 (4.2)	2 (9.5)	3 (6.7)
MI and CAD	21 (87.5)	19 (90.5)	40 (88.9)
Ischaemic stroke and CAD	1 (4.2)	3 (14.3)	4 (8.9)
PAD and CAD	4 (16.7)	4 (19.0)	8 (17.8)
MI and ischaemic stroke and CAD	1 (4.2)	3 (14.3)	4 (8.9)
LA sessions performed in the past 52 weeks [median (Q1, Q3)]			
	44 (41, 47)	44 (40, 46)	44 (41, 47)
Background LLT ^b^, *n* (%)			
Statin	23 (88.5)	22 (88.0)	45 (88.2)
Ezetimibe	15 (57.7)	15 (60.0)	30 (58.8)
Alirocumab	0	2 (8.0)	2 (3.9)
Evolocumab	5 (19.2)	0	5 (9.8)
Inclisiran	1 (3.8)	2 (8.0)	3 (5.9)
Bempedoic acid	0	1 (4.0)	1 (2.0)
Laboratory measurements			
Lp(a) (mg/dl) [median (Q1, Q3)]	90 (64, 109)	74 (66, 92)	84 (64, 97)
Lp(a) (nmol/l) [median (Q1, Q3)]	196 (139, 238)	156 (137, 199)	180 (137, 215)
Total cholesterol (mmol/l) [median (Q1, Q3)]	3.4 (2.9, 4.0)	3.4 (2.9, 3.7)	3.4 (2.9, 3.7)
LDL-C (mmol/l) [median (Q1, Q3)]	1.6 (1.2, 2.0)	1.5 (1.2, 1.9)	1.6 (1.2, 1.9)
HDL-C (mmol/L) [median (Q1, Q3)]	1.2 (1.1, 1.4)	1.2 (1.1, 1.6)	1.2 (1.1, 1.6)
Triglycerides (mmol/L) [median (Q1, Q3)]	1.3 (1.1, 1.6)	1.5 (1.0, 1.9)	1.4 (1.0, 1.7)
ApoB (mg/dl) [median (Q1, Q3)]	67 (56, 80)	62 (53, 69)	63 (53, 75)
HbA1c (%) [median (Q1, Q3)]	5.8 (5.5, 6.0)	5.6 (5.5, 5.9)	5.7 (5.5, 6.0)

^a^Percentages are based on the number of patients with ≥1 qualifying event category. Combinations of qualifying events with no reported occurrence are not shown.

^b^Prior medications (defined as those started prior to randomization visit).

ApoB, apolipoprotein B-100; BMI, body mass index; CS, clinically significant; CVD, cardiovascular disease; DBP, diastolic blood pressure; HDL-C, high-density lipoprotein cholesterol; LA, lipoprotein apheresis; LDL-C, low-density lipoprotein cholesterol; LLT, lipid-lowering therapy; Lp(a), lipoprotein(a); ms, mean sitting; SBP, systolic blood pressure; Q1/Q3, quartile 1/3.

### Efficacy

**Figure 2:**
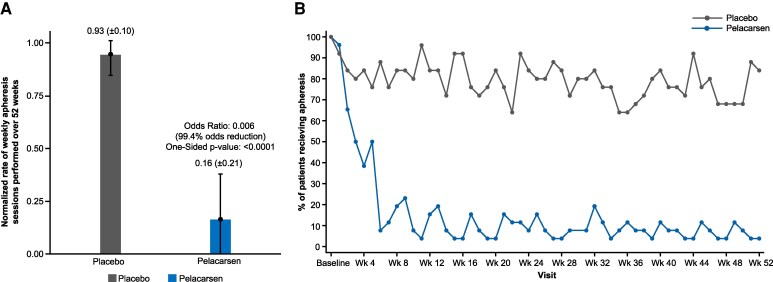
Rate of apheresis sessions over the double-blind 52-week period. *(A***)** Normalized rate of LA sessions was calculated by dividing the total number of LA sessions performed over the double-blind treatment period by the total weeks in the double-blinded treatment period, and is summarized in terms of mean (±SD) of all patients in both treatment arms using imputed data. Note: lines for ± SD were cut at 0.00 and 1.00, respectively. *(B*) Percentage of patients receiving LA during the double-blind period. LA, lipoprotein apheresis; SD, standard deviation; Wk, week

The mean (±SD) normalized rates of LA sessions performed over the double-blind period were 0.16 (±0.21) in the pelacarsen arm and 0.93 (±0.10) in the placebo arm. The odds ratio (95% CI) between treatment arms was 0.006 (0.003, 0.013), equivalent to a 99.4% reduction in the odds of requiring LA with pelacarsen compared to placebo (*P* < .0001) (*Figure 2A*). A summary of the proportion of patients receiving LA during the double-blind period is presented in *Figure 2B*. Supplementary data online, *Figure S2* shows the individual LA sessions that were performed or were not performed for each patients’ weekly visit throughout the study, including visits with missing LA data.

**Figure 3:**
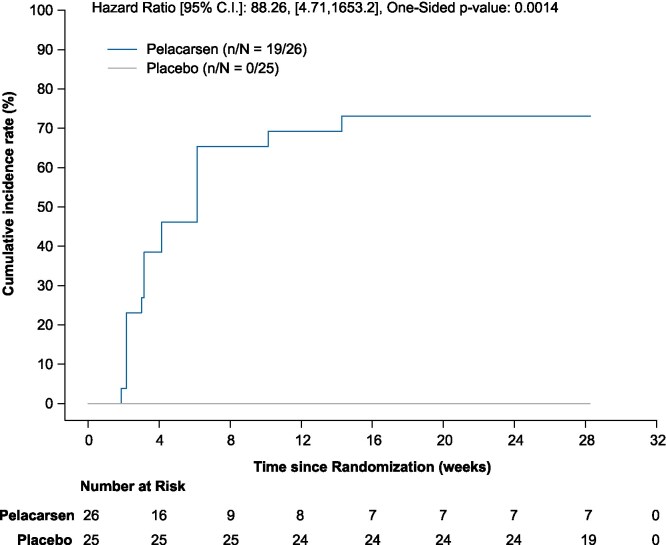
Time to achieve LA avoidance. In the placebo arm, six patients were censored up to week 28 (one patient died prior to week 12, and for 5 patients, their week 28 visit occurred slightly before or on week 28). In the pelacarsen arm, no patient was censored up to week 28. Median time (95% CI) to achieve LA avoidance with pelacarsen was 6.1 (3.1, 10.1) weeks and with placebo it was NE (NE, NE). Kaplan–Meier estimates were calculated based on imputed datasets, where the number at risk is the median of the number of patients at risk at each timepoint with the cumulative incidence rate depicting the probability of achieving LA. Data on incidence rates beyond week 28 were not possible as ≥24 weeks of consecutive LA avoidance, within the 52-week double-blind period, is required to achieve this endpoint. HRs (95% CI), along with the one-sided *P*-value, were derived from a Cox-regression model with treatment as a factor and log-transformed baseline Lp(a) as a covariate. Firth's penalized maximum likelihood approach was utilized. *n*/*N*; median of the number of patients achieving LA/total number of patients in the treatment arm, CI, confidence interval; HR, hazard ratio; LA, lipoprotein apheresis; NE: not evaluable

The hazard of achieving LA avoidance was 88 times higher with pelacarsen than placebo (hazard ratio [HR]: 88.26; 95% CI: 4.71, 1653.15; *P* = .0014), occurring in 19 patients (73.1%) vs none in the placebo arm. The median time (95% CI) to achieve LA avoidance in the pelacarsen arm was 6.1 (95% CI: 3.1, 10.1) weeks (*Figure 3*). Total LA avoidance defined as no LA required for 41 consecutive weeks from week 12 to week 52 was achieved by 18 patients (69.2%) in the pelacarsen arm compared to no patients in the placebo arm (odds ratio [OR]: 163.20; 95% CI: 7.66, 3477.25; *P* = .0005). Comparable results were observed when analysed from week 24 to week 52 as an exploratory endpoint (see Supplementary data online, *Table S3*). Overall, mean normalized LA rates and ORs for LA occurring between weeks 12–52, and weeks 24–52, were strikingly lower in the pelacarsen arm, compared to placebo (see Supplementary data online, *Table S4*).

**Figure 4:**
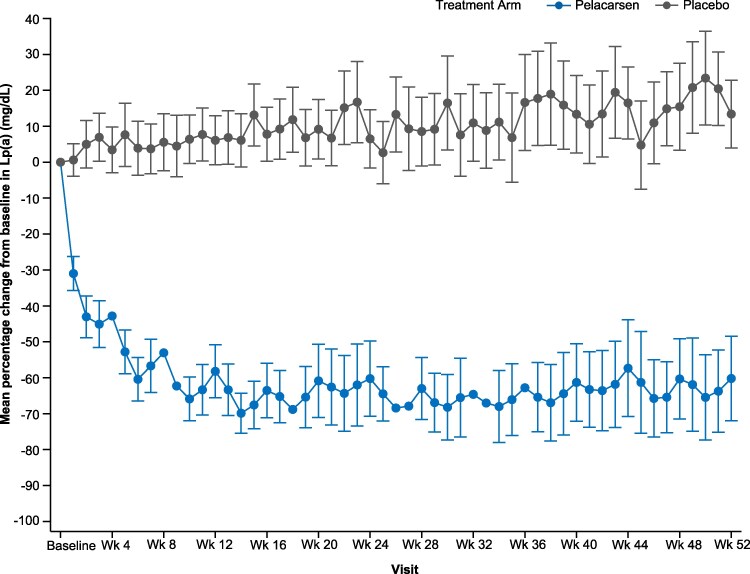
Mean percentage change in Lp(a) from baseline (mg/dL). Mean percentage change in Lp(a) from baseline with corresponding 95% CI for each weekly visit are presented. Lp(a) levels were measured pre-apheresis. CI, confidence interval; Lp(a), lipoprotein(a)

The geometric mean ratio of Lp(a) to baseline was 0.32 (95% CI: 0.26, 0.39) in the pelacarsen arm and 1.11 (95% CI: 0.89, 1.38) in the placebo arm. The ratio of geometric mean ratio (pelacarsen/placebo) of Lp(a) at week 52 was 0.28 (95% CI: 0.21, 0.39), equivalent to a *−*72% (95% CI: *−*79%, *−*61%) placebo-adjusted change from baseline, as measured in mg/dL (*P* < 0.0001). Using a similar analysis with Lp(a) levels measured in nmol/L, the placebo-adjusted Lp(a) change with pelacarsen was *−*77% (95% CI: *−*84%, *−*67%; *P* < .0001). The results were confirmed in a sensitivity analysis using a model with unequal variances and robust standard errors (see Supplementary data online, Appendix  *S3*). Four patients (two in each treatment group) had missing Lp(a) data at week 52. The mean (95% CI) change in Lp(a) levels in both treatment arms at each weekly visit (measured in mg/dL) is presented in *Figure 4,* while the time-averaged mean Lp(a) levels are in Supplementary data online, *Figure S3*. No relevant changes from baseline to week 52 were observed for LDL-C, time-averaged LDL-C, total cholesterol, HDL-C, non-HDL-C, VLDL-C, triglycerides, and ApoB (see Supplementary data online, *Table S5*).

High treatment adherence was observed with pelacarsen in this study. Overall, 13.1 (±2.7) and 12.7 (±3.7) out of a total of 14 doses (per patient) were administered in the pelacarsen and placebo treatment arms throughout the study, respectively.

### Safety

Overall, 96.1% of the patients experienced TEAEs (100% with pelacarsen and 92% with placebo). TEAEs and TESAEs are summarized in *Table 2*. TEAEs related to the study drug were reported in 18 patients (69.2%) receiving pelacarsen and 3 (12.0%) receiving placebo. These events were mostly injection site reactions, primarily mild, with one moderate event, occurring in 16 (61.5%) patients receiving pelacarsen and 3 (12.0%) receiving placebo. No TESAEs related to the study drug were reported in either treatment arm. Study drug interruptions due to TEAEs occurred in 2 (7.7%) patients treated with pelacarsen and 1 (4.0%) patient receiving placebo. Further information on study treatment interruptions can be found in Supplementary data online, *Table S2*. No patients from the pelacarsen arm discontinued the study due to TEAEs or TESAEs, whereas in the placebo arm, 2 (8.0%) patients discontinued due to a TEAE at Days 15 and 83 of the study, including 1 (4.0%) patient who discontinued due to a TESAE. All TEAE leading to discontinuation were reported as ‘not related’ by the investigators. One death was reported in the placebo arm due to lung carcinoma, which was assessed by the investigator as not related to the study treatment. The four most common TEAEs were nasopharyngitis (23.1% [*n* = 6] vs 36.0% [*n* = 9]), COVID-19 (23.1% [*n* = 6] vs 20.0% [*n* = 5]), injection site erythema (38.5% [*n* = 10] vs 0), and iron deficiency (7.7% [*n* = 2] vs 20.0% [*n* = 5]) when comparing pelacarsen vs placebo (*Table 2*). No severe TEAEs at the injection site were reported in either treatment arm (*Table 2*).

**Table 2:** Safety summary

	Pelacarsen *n* = 26 *n* (%)	Placebo *n* = 25 *n* (%)	Total *n* = 51 *n* (%)
Patients with ≥1 TEAE	26 (100)	23 (92.0)	49 (96.1)
Patients with ≥1 TEAE related to study drug	18 (69.2)	3 (12.0)	21 (41.2)
Patients with ≥1 TESAE	7 (26.9)	6 (24.0)	13 (25.5)
Patients with ≥1 TESAE related to study drug	0	0	0
Patients who interrupted study drug due to TEAE	2 (7.7)	1 (4.0)	3 (5.9)
Patients who interrupted study drug due to TESAE	0	1 (4.0)	1 (2.0)
Patients who discontinued study drug due to TEAE	0	2 (8.0)	2 (3.9)
Patients who discontinued study drug due to TESAE	0	1 (4.0)	1 (2.0)
Patients who died	0	1 (4.0)	1 (2.0)
Most common TEAEs (≥10%)			
Nasopharyngitis	6 (23.1)	9 (36.0)	15 (29.4)
COVID-19	6 (23.1)	5 (20.0)	11 (21.6)
Injection site erythema	10 (38.5)	0	10 (19.6)
Iron deficiency	2 (7.7)	5 (20.0)	7 (13.7)
Back pain	4 (15.4)	2 (8.0)	6 (11.8)
Fatigue	5 (19.2)	1 (4.0)	6 (11.8)
Headache	6 (23.1)	0	6 (11.8)
Injection site pruritus	4 (15.4)	1 (4.0)	5 (9.8)
Respiratory tract infection	5 (19.2)	0	5 (9.8)
Arthralgia	3 (11.5)	1 (4.0)	4 (7.8)
Injection site haematoma	3 (11.5)	1 (4.0)	4 (7.8)
Influenza like illness	3 (11.5)	0	3 (5.9)
Injection site rash	3 (11.5)	0	3 (5.9)

TEAE refers to an event starting after the first dose of study drug and up to 16 weeks (112 days) after the date of the last study drug injection, the death of the patient, or the patients study end date, whichever comes first. A patient with multiple occurrences of a TEAE under one treatment is counted only once in this TEAE category for that treatment. Preferred terms are presented in descending frequency in the total column.

TEAE, treatment-emergent adverse event; TESAE, treatment-emergent serious adverse event.

## Discussion

Weekly LA, although effective in acutely lowering Lp(a) levels, imposes a substantial burden on patients’ daily lives. Each session typically lasts several hours and must be conducted at specialized centres, requiring patients to commit significant time towards travel, treatment, and recovery. The invasive nature of the procedure may also contribute to physical discomfort and emotional fatigue. These challenges can negatively impact adherence and overall quality of life, particularly in patients undergoing LA for extended periods.^10^

In the Lp(a)FRONTIERS APHERESIS trial, treatment with pelacarsen 80 mg every 4 weeks significantly reduced the odds of requiring LA in patients with established CVD undergoing weekly LA for elevated Lp(a) by more than 99%. The majority of patients achieved long-lasting avoidance of LA and sustained lowering of Lp(a) by more than 70% from study baseline (*Structured Graphical Abstract*).

The decision to perform LA in this study was based on Lp(a) levels of >60 mg/dl at the prior visit, and the use of pelacarsen to reduce Lp(a) translated into significantly less patients requiring LA throughout the study (see Supplementary data online, *Figure S2*). Following a single dose of pelacarsen, approximately 40% of patients entered a period where at least 24 consecutive weeks of LA avoidance until week 52 were achieved (*Figure 3*).

Eight patients treated with pelacarsen did not achieve the secondary endpoint of ‘total LA avoidance’ (i.e. 41 consecutive weeks between weeks 12 and 52 without LA), mostly due to some individual visits in which Lp(a) was slightly above >60 mg/dl. Still, the normalized rate of LA performed between weeks 12 and 52 was strikingly lower in the pelacarsen arm (mean ± SD: 0.11 ± 0.23) vs placebo (mean ± SD: 0.93 ± 0.10) as shown in Supplementary data online, *Table S4*.

Throughout this 52-week study period, pelacarsen led to a profound and sustained reduction in Lp(a) levels compared to placebo, with placebo-adjusted changes from baseline of *−*72% and *−*77% when measured in mg/dl and nmol/l, respectively. Notably, this percentage change is relative to baseline Lp(a) levels, which were measured after at least 1 year of ongoing Lp(a)-lowering treatment with LA. While not investigated in this study, it is presumed that treatment-naïve Lp(a) levels are likely 20%–30% higher than reported baseline levels because weekly LA prevented the complete rebound of Lp(a) concentration. Therefore, the Lp(a)-lowering effect of 80 mg pelacarsen in treatment-naïve patients may be greater than that observed in the present study. This finding supports the dose selected for the ongoing Lp(a)HORIZON trial, which is investigating the effect of pelacarsen on cardiovascular outcomes in more than 8300 enrolled patients with elevated Lp(a) and established CVD^20^ (ClinicalTrials.gov: NCT04023552).

The incidence of TEAEs was generally balanced between pelacarsen and placebo treatment arms, apart from injection site reactions, which occurred in 61.5% of patients in the pelacarsen arm compared to 12.0% in the placebo arm. These injection site reactions were predominantly mild, consistent with observations from the Phase 2b trial and commonly reported for subcutaneously administered antisense oligonucleotides.^16,21^ The most commonly reported injection site reactions in this study were injection site erythema (38.5% vs 0%), injection site pruritus (15.4% vs 4.0%), injection site rash (11.5% vs 0%), and injection site haematoma (11.5% vs 4.0%) for pelacarsen and placebo, respectively. No patients in either treatment arm discontinued the study due to TEAEs or experienced a TESAE related to the study drug. Pelacarsen’s good tolerability is further supported by high treatment adherence to the blinded study medication.

LA is established as an effective approach that acutely lowers Lp(a) by up to 75%. As Lp(a) synthesis is not suppressed by LA, Lp(a) levels gradually rebound, and time-averaged reductions range between 25% and 40%, dependent on the frequency of LA (i.e. weekly or biweekly).^12,22^

This study has several limitations: First, due to its small sample size and relatively short duration, this study is neither suited to establishing the long-term safety profile of pelacarsen, nor to evaluate the impact of pelacarsen treatment on cardiovascular outcomes. In this regard, the ongoing Lp(a)HORIZON cardiovascular outcomes trial will provide more information, based on >15 000 patient-years exposure to pelacarsen by end-of-study.^20^ It is also important to note that in this study, sparse data bias may have contributed to the fairly pronounced results obtained when applying Firth’s penalized maximum likelihood approach, i.e. the HR for time to LA avoidance and the OR for achieving total LA avoidance.^23^

Second, despite adequate blinding of all participating parties, the occurrence or omission of LA may have unintentionally indicated the treatment allocation to some study participants or investigators. While the use of a sham apheresis procedure was considered as a potential method to strengthen blinding, this approach was ultimately deemed ethically unacceptable due to the physical burden and associated safety risks of simulating an invasive procedure without clinical benefits. Importantly, both primary and secondary endpoints in the study were objective and protocol-defined, based on the occurrences of LA sessions, which were strictly determined by blinded Lp(a) levels measured centrally. Given the objective nature of these endpoints and their dependence on blinded Lp(a) measurements, the integrity and robustness of the data in the authors’ view remains intact despite the inherent challenges in maintaining full blinding.

Third, given the very specific patient population targeted in this study (i.e. all patients under an established weekly LA regimen), the extrapolation of the observed treatment effect on Lp(a) lowering to LA-naïve patients with elevated Lp(a) is limited, and the effect size reported here potentially underestimates the true effect size. Similarly, the size of this single-country study is not suited to evaluating the generalizability of findings across diverse ethnic and racial groups. This will be adequately addressed in the Lp(a)HORIZON study that is currently ongoing in 42 countries across all continents.

Finally, given the high financial burden of frequent LA (approximately 50 000 EUR per year in Germany) and the high prevalence of elevated Lp(a), the future cost-effectiveness of pelacarsen is of great interest for healthcare systems. As pelacarsen is an investigational drug with the submission-enabling pivotal Phase 3-study Lp(a)HORIZON still ongoing, no pricing or cost-effectiveness estimations are currently available.

The findings of Lp(a)FRONTIERS APHERESIS study demonstrate that pelacarsen is a highly effective Lp(a)-targeted therapy, substantially reducing the burden associated with frequent LA in this high-risk population with elevated Lp(a) and established CVD. Taken together with the fact that pelacarsen was generally well tolerated, these data suggest that pelacarsen could replace LA in patients who specifically require this procedure to manage their elevated Lp(a).

## Supplementary Material

ehag073_Supplementary_Data

## Declarations

### Data Availability

Novartis is committed to sharing with qualified external researchers, patient-level data and supporting clinical documents from eligible studies. These requests are reviewed and approved by an independent review panel on the basis of scientific merit. All data provided is anonymized to respect the privacy of patients who have participated in the trial in line with applicable laws and regulations. This trial data availability is according to the criteria and process described on www.clinicalstudydatarequest.com.

### Pre-registered Clinical Trial Number

ClinicalTrials.gov, identifier: NCT05305664.
