# Supplementary material for: Pelacarsen and lipoprotein(a) apheresis in secondary prevention: the Lp(a)FRONTIERS APHERESIS trial
Source: Eur Heart J. 2026 Feb 21;47(25):3284–94. doi: 10.1093/eurheartj/ehag073 (PMC13318419; doi:10.1093/eurheartj/ehag073)
Supplement: ehag073_Supplementary_Data [file ehag073_supplementary_data.zip › Supplemental_APHERESIS resubmission file_10February2026_clean.docx]

Pelacarsen and lipoprotein(a) apheresis in secondary prevention:

the Lp(a)FRONTIERS APHERESIS trial

**Supplemental file**

# Contents

[Contents 1](#_Toc214614160)

[**Appendix 1. Protocol amendment for qualifying cardiovascular conditions and updated baseline assumptions** 2](#_Toc214614161)

[**Appendix 2. Measurement of Lp(a)** 2](#_Toc214614162)

[**Appendix 3: Details of multiple imputation and sensitivity analysis for Lp(a) change from baseline at week 52** 2](#_Toc214614163)

[**Table S1. Full inclusion and exclusion criteria for Lp(a)FRONTIERS APHERESIS** 4](#_Toc214614164)

[**Table S2. Duration of exposure** 7](#_Toc214614165)

[**Table S3. Total lipoprotein avoidance achieved between week 12 and week 52, and week 24 and week 52** 8](#_Toc214614166)

[**Table S4. Mean rates of apheresis between week 12 and week 52, and week 24 and week 52** 8](#_Toc214614167)

[**Table S5. Change in lipid profile parameters** 9](#_Toc214614168)

[**Figure S1. Study design** 10](#_Toc214614169)

[**Figure S2. Lipoprotein apheresis sessions per patient performed during the double-blind period** 11](#_Toc214614170)

[**Figure S3. Time-averaged mean Lp(a) levels (mg/dL)** 12](#_Toc214614171)

# **Appendix 1. Protocol amendment for qualifying cardiovascular conditions and updated baseline assumptions**

The study protocol was amended during enrolment to allow participation to patients with clinically significant coronary artery disease, but without a prior MI, aiming for a diverse study population that reflects the LA-population in Germany. The plausible range of apheresis rates to be expected in the placebo arm was also reassessed, leading to a reduced sample size requirement from initially 60 to 46.

# **Appendix 2. Measurement of Lp(a)**

Lp(a) concentrations as used for the secondary and exploratory endpoint-analyses were measured in both mg/dL and nmol/L using the Roche Tina-Quant assay using Medpace as central laboratory. Lp(a) levels that informed the decision on apheresis-conduct were measured using the Randox mass assay. This choice was driven by logistical considerations, as Randox samples (processed in the Netherlands) achieved a shorter turnaround time that was necessary to inform the weekly apheresis sessions.

# **Appendix 3: Details of multiple imputation and sensitivity analysis for Lp(a) change from baseline at week 52**

**Missing data of lipoprotein apheresis session**

1. If at the previous visit, the Lp(a) value was > 60 mg/dL, the missing lipoprotein apheresis was imputed as having occurred; otherwise, as not having occurred.
2. In case of study discontinuation, missed visits after discontinuation were imputed as apheresis having occurred. For patients who died, only the data up to death were used (“while alive”).

Note: if the Lp(a) value was missing for 1), then the imputed value was used.

**Missing Lp(a) data**

A two-step algorithm to impute the missing data was employed. following the ideas outlined by Carpenter et al (2013) using an approximate Bayesian model assuming missingness-at-random (MAR).

Step 1:

- A mixed model for repeated measures (MMRM) was fitted to all the data observed prior to the pre-defined intercurrent events (study drug discontinuation, change in permitted concomitant medication, death). This modeled the joint distribution of each study participant’s Lp(a) at different visits through a multivariate normal distribution with a single AR (1) covariance matrix across study arms, including study treatment, baseline Lp(a), and study treatment by visit interaction.
- 100 estimates of parameters from the MMRM were obtained by repeatedly applying restricted maximum-likelihood (REML) estimation of the MMRM model to nonparametric bootstrap samples. The bootstrap samples were obtained through resampling stratified by study arm from the original dataset.

Step 2:

- For each of the 100 parameter estimates from Step 1, for each study participant with missing Lp(a) data, the joint distribution of observed and missing data $(Y_{obs}, Y_{miss})\sim MN(\tilde{\mu},\tilde{\Sigma})$ with mean $\tilde{\mu}=(\tilde{\mu}_{obs}, \tilde{\mu}_{miss})$ and covariance matrix $\tilde{\Sigma}$ was constructed. The mean vector $\tilde{\mu}$ was chosen to reflect the imputation strategy, i.e., MAR.
- Then, the missing Lp(a) data was imputed by drawing from the conditional distribution ${Y_{miss}|Y}_{obs}$ of missing data given the observed values, providing 100 complete data sets of Lp(a) values.

**Sensitivity analysis for Lp(a) change from baseline at week 52**

A sensitivity analysis was conducted using unequal variances between the treatment groups and robust standard errors (for each imputed dataset). The estimates were combined via Rubin’s rule and confirmed the original analysis with the following results for the placebo-adjusted change from baseline:

mg/dL: *−*72% (95% CI: *−*78%, *−*63%; p<0.0001)

nmol/L: *−*77% (95% CI: *−*83%, *−*68%; p<0.0001)

# **Table S1. Full inclusion and exclusion criteria for Lp(a)FRONTIERS APHERESIS**

| **Inclusion criteria** |
| --- |
| 1. Signed informed consent obtained prior to study participation |
| 1. Male and female ≥18 to ≤80 years of age at screening |
| 1. Patients currently undergoing isolated Lp(a) apheresis on a weekly schedule in Germany for ≥12 months prior to screening with ≥35 sessions within the past 52 weeks prior to randomization |
| 1. Lp(a) >60 mg/dL at screening visit week 2 |
| 1. Stable lipoprotein apheresis technique, volume and duration within 3 months prior to screening and no planned changes to lipoprotein apheresis parameters during the trial |
| 1. Established CVD defined as any of the following:  - Spontaneous prior MI (either ST-elevation MI or non-ST-elevation MI), which was not the result of PCI or CABG, and which occurred in the period ≥3 months prior to the screening visit. Confirmation of MI was a patient history/patient recollection of signs and symptoms consistent with presentation of MI, and at least one of the qualifying criteria* - History of ischemic stroke (an acute episode of focal cerebral, spinal, or visual dysfunction caused by infarction of central nervous system tissue) having occurred in the period ≥3 months prior to the screening visit documented by CT scan, MRI or other visualisation method. TIA, lacunar infarction or embolic stroke (not of atherosclerotic origin) were not qualifying events - Clinically significant symptomatic PAD evidenced by intermittent claudication with an ABI ≤0.90 and/or limb amputation or revascularisation due to lower limb ischemia. Thromboangiitis obliterans is not a qualifying event - History of clinically significant symptomatic CAD, evidenced by either typical stable angina pectoris symptoms under mild/moderate exertion (Canadian Cardiovascular Society-class IV), which required either interventional or surgical coronary revascularisation |
| **Exclusion criteria** |
| 1. Uncontrolled hypertension defined as sitting SBP ≥160 mmHg and/or DBP ≥100 mmHg (mean of 3 measurements for each assessment) at the screening visit |
| 1. Treatment with stable dose of a PCSK9 inhibitor (evolocumab, alirocumab) for <12 weeks before randomization |
| 1. Between screening visit and randomization visit (Day 1): MI, stroke, coronary or lower limb revascularisation, major cardiac or non-cardiac surgery. The participant can be re-screened 3 months after the relevant event/procedure |
| 1. Treatment with niacin ≥2000 mg QD in the 3 months before the screening visit; niacin in multi-vitamins is allowed |
| 1. Planned or expected cardiac, cerebrovascular or peripheral artery surgery or coronary revascularisation |
| 1. Heart failure NYHA class IV at screening or randomization visit |
| 1. History of haemorrhagic stroke or other major bleeding, or if occurring between screening and randomization visit |
| 1. Severe concomitant non-CVD that was expected to reduce life expectancy to <5 years, at screening or randomization visit |
| 1. Known active severe infection or major haematologic, metabolic, gastrointestinal or endocrine dysfunction in the judgement of the investigator, at screening or randomization visit |
| 1. History of malignancy of any organ system (other than localised basal cell carcinoma or squamous cell carcinoma of the skin, or in situ cervical cancer), treated or untreated, within the past 5 years, or if diagnosed between screening and randomization visit, regardless of whether there is evidence of local recurrence or metastases |
| 1. Platelet count <140,000 per mm^3^ from central laboratory test at screening visit, confirmed by a second central laboratory test prior to the randomization visit |
| 1. eGFR ≤30 mL/min/1.73m^2^ from central laboratory test at screening visit, confirmed by a second central laboratory test prior to the randomization visit; or patient on dialysis |
| 1. Significant glomerular disease (including but not limited to IgA nephropathy, diabetic nephropathy, systemic lupus erythematosus, etc) with urinary PCR >500 mg/g (56.6 mg/mmol) at screening visit, confirmed by a second central laboratory test prior to the randomization visit |
| 1. Active liver disease or hepatic dysfunction, defined as AST or ALT ≥2 times ULN from central laboratory test at screening visit, confirmed by a second central laboratory test prior to the randomization visit |
| 1. Total bilirubin ≥1.5 times the ULN from central laboratory test at screening visit, or patients with known Gilbert syndrome, independent of the total bilirubin value at screening visit |
| 1. Positive HIV, hepatitis C screening or hepatitis B surface antigen tests from central laboratory test at screening visit |
| 1. Any other conditions, at screening visit or between screening and randomization visit, which in the opinion of the investigator would make the patient unsuitable for inclusion, or could interfere with the patient participating in or completing the study |
| 1. Treatment with an oligonucleotide or siRNA within 9 months before screening visit. Treatment with inclisiran if first injection occurred within 30 days prior to randomization |
| 1. History of hypersensitivity to any of the study treatments or its excipients or to drugs of similar chemical classes |
| 1. Use of other investigational drugs within 5 half-lives of screening visit or within 30 days, whichever was longer |
| 1. Unwillingness or inability (e.g. physical or cognitive) to comply with study procedures and schedule |
| 1. Pregnant or nursing (lactating) women |
| 1. Women of childbearing potential, defined as all women physiologically capable of becoming pregnant, unless they are using highly effective methods of contraception** while taking study treatment and for 16 weeks after the last dose of the investigational drug |
| *Documentation of cardiac biomarkers that exceed the diagnostic threshold of a local laboratory for MI, pathological Q waves on ECG or other ECG changes (ST elevation, ST-depression and T wave changes or pathological Q waves), imaging evidence of loss of viable myocardium or regional wall motion abnormality in a pattern consistent with an infarction or ischemic aetiology, or identification of a coronary thrombus by angiography at the time of presentation with MI.  ******Highly effective contraception methods included (i) total abstinence (periodic abstinence and withdrawal were not acceptable methods of contraception); (ii) female sterilisation, total hysterectomy or bilateral tubal ligation ≥6 weeks before taking study treatment. In case of oophorectomy alone, only when the reproductive status of the woman was confirmed by follow up hormone assessment; (iii) male sterilisation (≥6 months prior to screening). For female participants on the study, the vasectomised male partner should be the sole partner for that participant; and (iv) use of oral, injected, or implanted hormonal methods of contraception or placement of an IUD or IUS, or other forms of hormonal contraception that have comparable efficacy (failure rate <1%), for example hormone vaginal ring or transdermal hormone contraception. In case of use of oral contraception, women should have been stable on the same pill for ≥3 months prior to administration of study treatment.  ABI, ankle-brachial index; ALT, alanine aminotransferase; AST, aspartate aminotransferase; CABG, coronary artery bypass graft; CAD, coronary artery disease; CT, computed tomography; CVD, cardiovascular disease**;** DBP, diastolic blood pressure; ECG, electrocardiogram; eGFR, estimated glomerular filtration rate; HIV, human immunodeficiency virus; IgA, immunoglobulin A**;** IUD, intrauterine device; IUS, intrauterine system; Lp(a), lipoprotein(a); MI, myocardial infarction; MRI, magnetic resonance imaging; NYHA, New York Heart Association; PAD, peripheral artery disease; PCI, percutaneous coronary intervention; PCR, protein-creatinine ratio; PCSK9, proprotein convertase subtilisin/kexin type 9; QD, once daily; SBP, systolic blood pressure; siRNA, small interfering ribonucleic acid; TIA, transient ischemic attack; ULN, upper limit of normal. |

# **Table S2. Duration of exposure**

| **Exposure** | **Pelacarsen n=26** | **Placebo n=25** | **Total N=51** |
| --- | --- | --- | --- |
| **Duration of double-blind period (days), mean (±SD)** | 362.7 (±16.20) | 348.0 (±61.36) | 355.5 (±44.64) |
| **Total duration of double-blind period (patient-years)** | 25.8 | 23.8 | 49.6 |
|  | | | |
| **Duration on treatment period (days), mean (±SD)** | 355.7 (±36.10) | 337.9 (±77.11) | 347.0 (±59.88) |
| **Total duration on treatment period (patient-years)** | 25.3 | 23.1 | 48.4 |
|  | | | |
| **Duration of exposure (days),  mean (±SD)** | 345.5 (±56.57) | 337.9 (±77.11) | 341.8 (±66.85) |
| **Total duration of exposure  (patient-years)** | 24.5 | 23.1 | 47.6 |
|  | | | |
| **At least one study treatment interruption*, n (%)** | 3 (11.5) | 1 (4.0) | 4 (7.8) |
| **Reason for study treatment interruption*, n (%)** | | | |
| **Adverse Event** | 2 (7.7) | 0 | 2 (3.9) |
| **Physician Decision** | 1 (3.8) | 1 (4.0) | 2 (3.9) |
| **Subject Decision** | 2 (7.7) | 0 | 2 (3.9) |
| **Number of study treatment interruption episodes**, n (%)** | | | |
| **None** | 24 (92.3) | 25 (100) | 49 (96.1) |
| **1** | 1 (3.8) | 0 | 1 (2.0) |
| **2** | 1 (3.8) | 0 | 1 (2.0) |
| **Duration of study treatment interruption episodes** (weeks)** | | | |
| **Median** | 19.0 | 0 | 19.0 |
| **Q1 - Q3** | 8.9 - 29.0 | 0 | 8.9 - 29.0 |

Duration of double-blind period in days is defined as: date of last known visit on study − date of first dose of treatment +1. Duration on-treatment period in days defined as: the time from the first dose date to the earliest date of the following: the last dose date +112, patient’s death date, or patient’s study end date. Patient-years of double-blind period for each patient is calculated as: (Duration of double-blind period in days)/365.25. Duration of exposure is defined as: duration of on-treatment period minus duration of study treatment interruption episodes.
*Study treatment interruption is the case where the dose was recorded as interrupted in the CRF. Subjects with more than one treatment interruption were counted multiple times for each unique reason; ** Any study treatment interruption interval >60 days, calculated as date of current injection date − date of previous injection, is considered as a treatment interruption episode. Duration of study treatment interruption episodes are defined as the sum of all intervals of study treatment interruption episodes.
CRF, case report form; Q1/3, quartile 1/3.

# **Table S3. Total lipoprotein avoidance achieved between week 12 and week 52, and week 24 and week 52**

|  | **Pelacarsen n=26** | **Placebo n=25** | **Pelacarsen vs placebo** | |
| --- | --- | --- | --- | --- |
| Timepoint | n (%) | n (%) | Odds Ratio  (95% CI) | One-sided  p-value |
| Week 12 to Week 52 | 18 (69.23) | 0 | 163.20  (7.66, 3477.25) | 0.0005 |
| Week 24 to Week 52 | 19 (73.08) | 0 | 200.93  (9.13, 4423.17) | 0.0004 |

n is the median of the total number of patients with total apheresis avoidance from either week 12 or week 24 to week 52 from imputed datasets. N is the total number of patients in the analysis population. Total apheresis avoidance has been analyzed using a logistic regression model with treatment as a factor and log-transformed baseline Lp(a) as a covariate.

CI, confidence interval; Lp(a), lipoprotein(a).

# **Table S4. Mean rates of apheresis between week 12 and week 52, and week 24 and week 52**

|  | **Pelacarsen n=26** | **Placebo n=25** | **Pelacarsen vs placebo** | |
| --- | --- | --- | --- | --- |
| Timepoint | Mean (SD) | Mean (SD) | Odds ratio  (95% CI) | One-sided  p-value |
| Week 12 to Week 52 | 0.11  (0.23) | 0.93  (0.10) | 0.003  (0.001, 0.011) | <0.0001 |
| Week 24 to Week 52 | 0.10  (0.24) | 0.93  (0.10) | 0.003  (0.001, 0.015) | <0.0001 |

Rate (proportion) of apheresis sessions is calculated with imputed data using the following formula: total number of apheresis sessions performed from either week 12 or week 24 to week 52/total corresponding weeks from week 12 or week 24 to week 52 and is summarized in terms of mean (±SD) across all patients per treatment arm. Conditional odds ratio and its 95% CI are from a fractional regression model with logistic function, including treatment as a fixed factor and log-transformed baseline Lp(a) as a covariate.
CI, confidence interval; Lp(a), lipoprotein(a);

# **Table S5. Change in lipid profile parameters**

|  | **Median (Q1, Q3) value at baseline** | | **Median (Q1, Q3) value at Week 52** | |
| --- | --- | --- | --- | --- |
| **Lipid parameter** | **Pelacarsen** | **Placebo** | **Pelacarsen** | **Placebo** |
| LDL-C (mmol/L)* | 1.6 (1.2, 2.0) | 1.5 (1.2, 1.9) | 1.7 (1.0, 2.0) | 1.6 (1.3, 1.9) |
| LDL-C (mmol/L)^†^ | 1.3 (1.0, 1.6) | 1.2 (1.1, 1.5) | 1.7 (1.0, 1.9) | 1.3 (1.1, 1.6) |
| Total cholesterol (mmol/L) | 3.4 (2.9, 4.0) | 3.4 (2.9, 3.7) | 3.6 (2.8, 4.3) | 3.3 (2.9, 4.0) |
| HDL-C (mmol/L) | 1.2 (1.1, 1.4) | 1.2 (1.1, 1.6) | 1.4 (1.1, 1.7) | 1.3 (1.0, 1.6) |
| Non-HDL-C (mmol/L) | 2.0 (1.8, 2.5) | 1.9 (1.6, 2.4) | 2.0 (1.4, 2.6) | 1.9 (1.8, 2.4) |
| VLDL-C (mmol/L) | 0.6 (0.5, 0.7) | 0.7 (0.4, 0.9) | 0.5 (0.4, 0.8) | 0.7 (0.4, 0.8) |
| Triglycerides (mmol/L) | 1.3 (1.1, 1.6) | 1.5 (1.0, 1.9) | 1.2 (1.0, 2.2) | 1.5 (0.9, 1.8) |
| ApoB (mg/dL) | 67 (56, 80) | 62 (53, 69) | 66 (51, 75) | 64 (52, 77) |
| *Measured pre-apheresis  ^†^Time-averaged LDL-C, calculated using the Kroon formula CAVG = CMIN + 0.73(CMAX – CMIN). CMAX and CMIN are the immediate  pre and post apheresis LDL-C levels. If post apheresis LDL-C is missing due to apheresis avoidance, then the pre apheresis LDL-C is considered as time-averaged LDL-C.  ApoB, apolipoprotein B, HDL-C, high-density lipoprotein cholesterol; LDL-C, low-density lipoprotein cholesterol; VLDL-C, very-low density lipoprotein cholesterol. | | | | |

# **Figure S1. Study design**


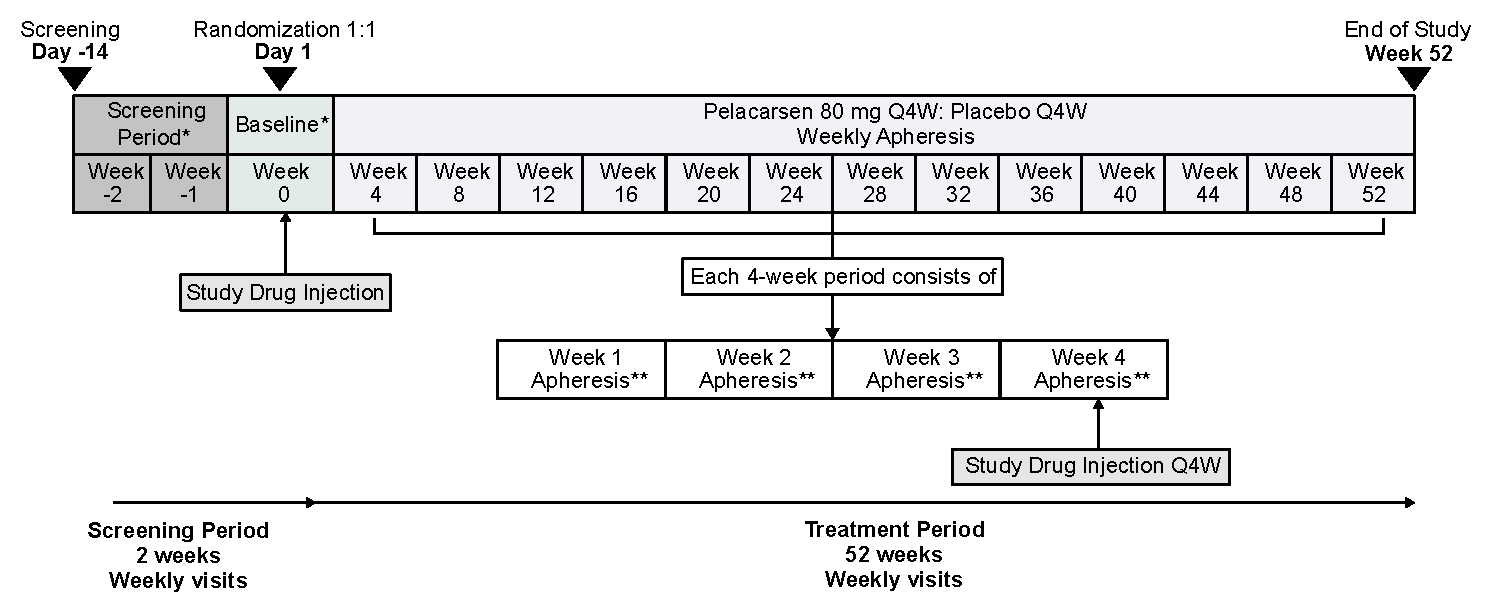


*QW apheresis was performed during the screening period and at baseline visit;
**Apheresis was only performed from Week 1 if the Lp(a) level was >60 mg/dL at the previous visit (N−1)
Lp(a), lipoprotein(a); QW, once weekly; Q4W, every 4 weeks.

# **Figure S2. Lipoprotein apheresis sessions per patient performed during the double-blind period**


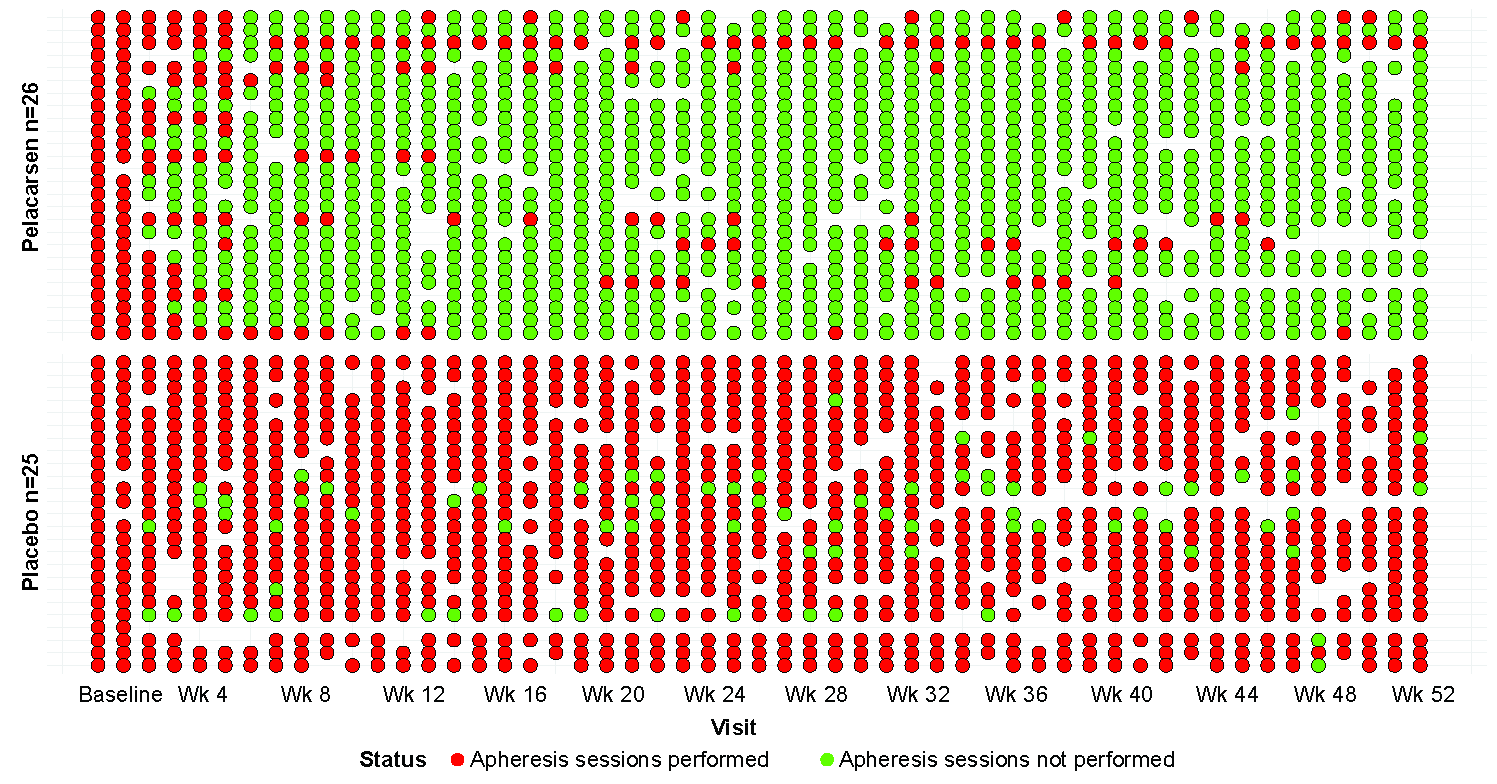


Blanks represent missing apheresis data at the visit. Apheresis sessions represent observed data.
Wk, week.

# **Figure S3.** **Time-averaged mean Lp(a) levels (mg/dL)**


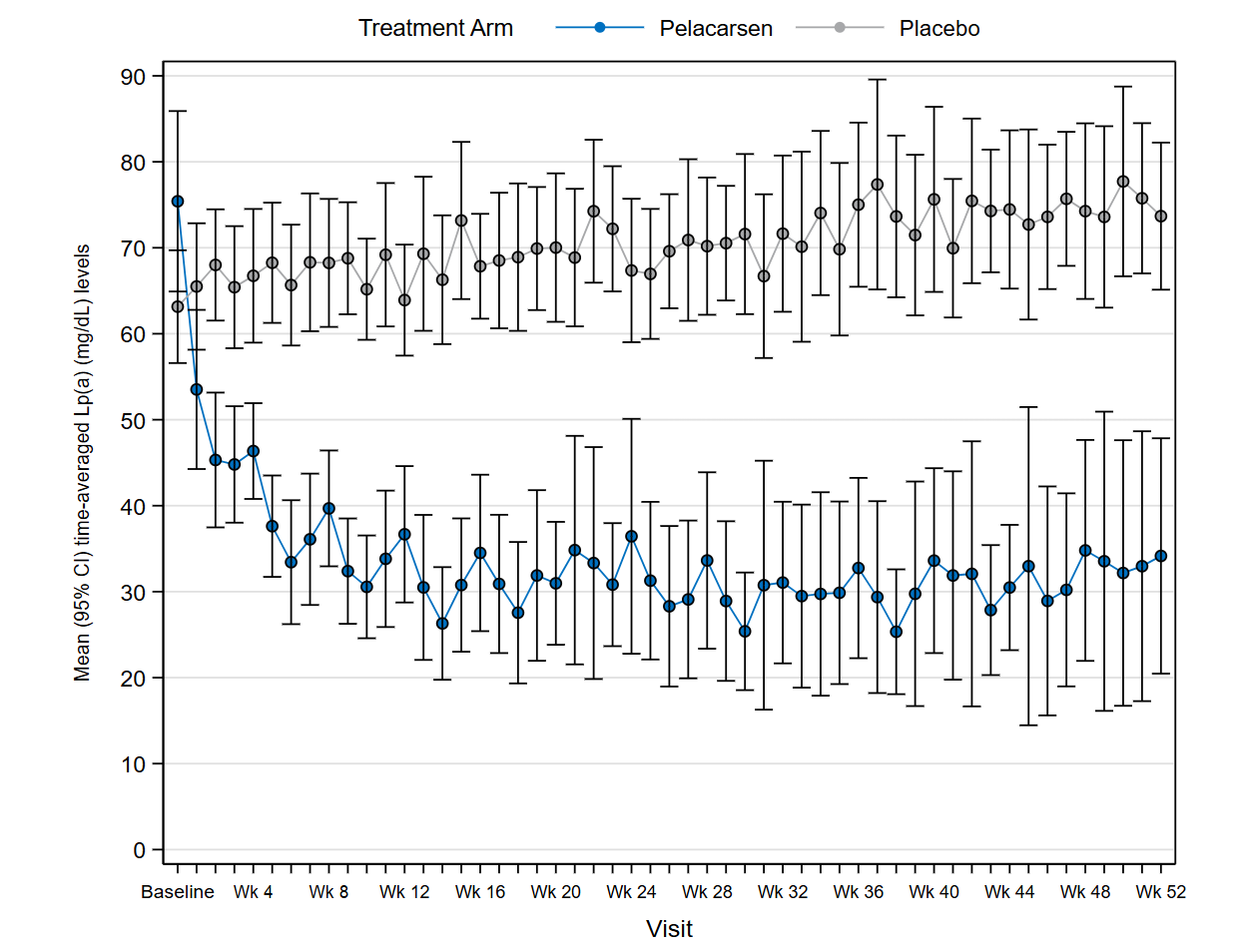


Time-averaged Lp(a)-values given were calculated using the Kroon-formula (CAVG = CMIN + 0.73 × (CMAX-CMIN), where CMAX and CMIN are the immediate pre- and post-apheresis Lp(a) levels ([Kroon AA, et al. Atherosclerosis. 2000;152(2):519-26](https://pubmed.ncbi.nlm.nih.gov/10998482/)).

CI, confidence interval; Lp(a), lipoprotein(a).
