# Supplementary figures and images for: Pelacarsen and lipoprotein(a) apheresis in secondary prevention: the Lp(a)FRONTIERS APHERESIS trial
Source: Eur Heart J. 2026 Feb 21;47(25):3284–94. doi: 10.1093/eurheartj/ehag073 (PMC13318419; doi:10.1093/eurheartj/ehag073)

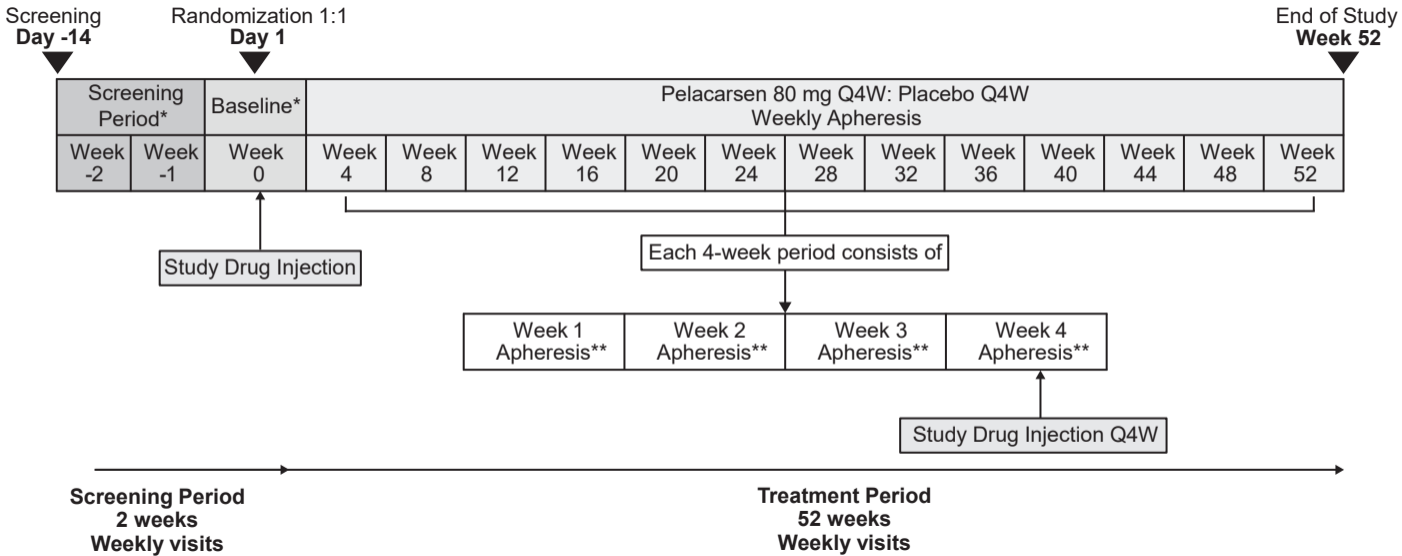

Supplement: ehag073_Supplementary_Data [file ehag073_supplementary_data.zip › Figure S1. Study design.pdf]

Pelacarsen n=26

Placebo n=25

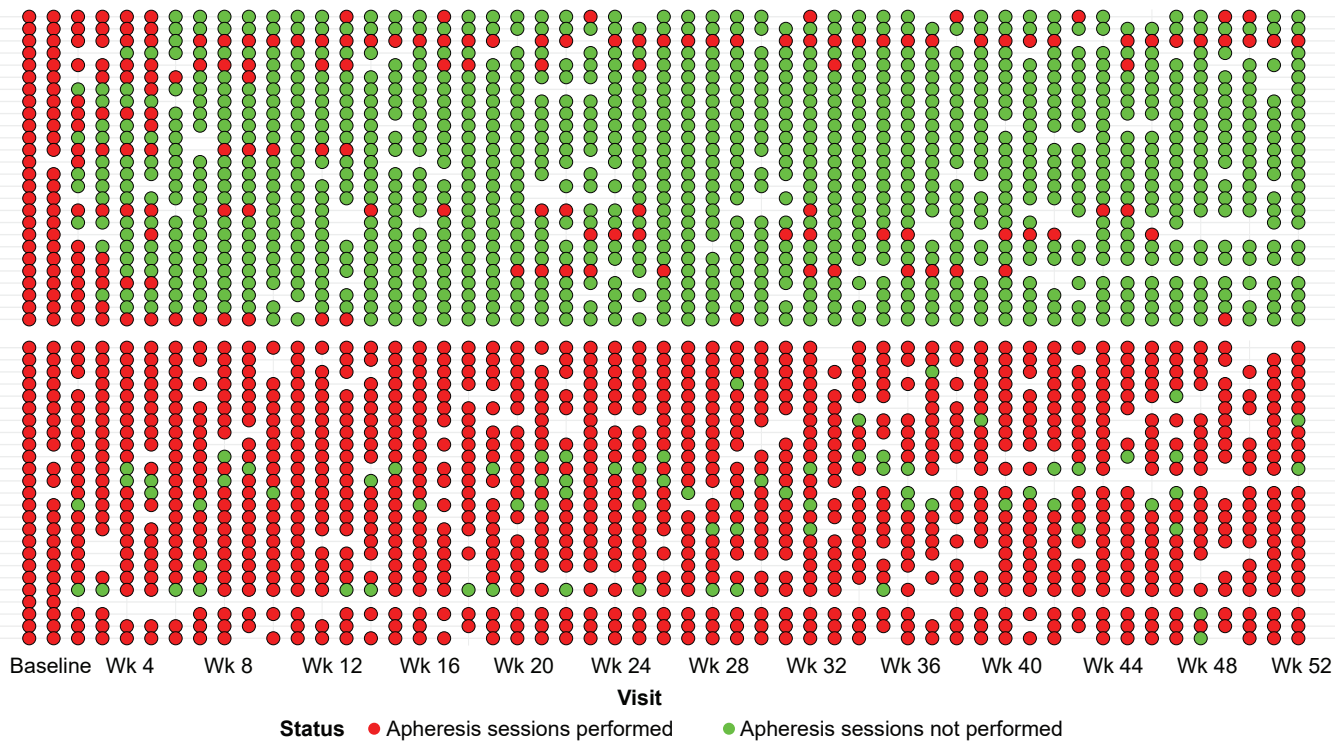

Supplement: ehag073_Supplementary_Data [file ehag073_supplementary_data.zip › Figure S2..pdf]

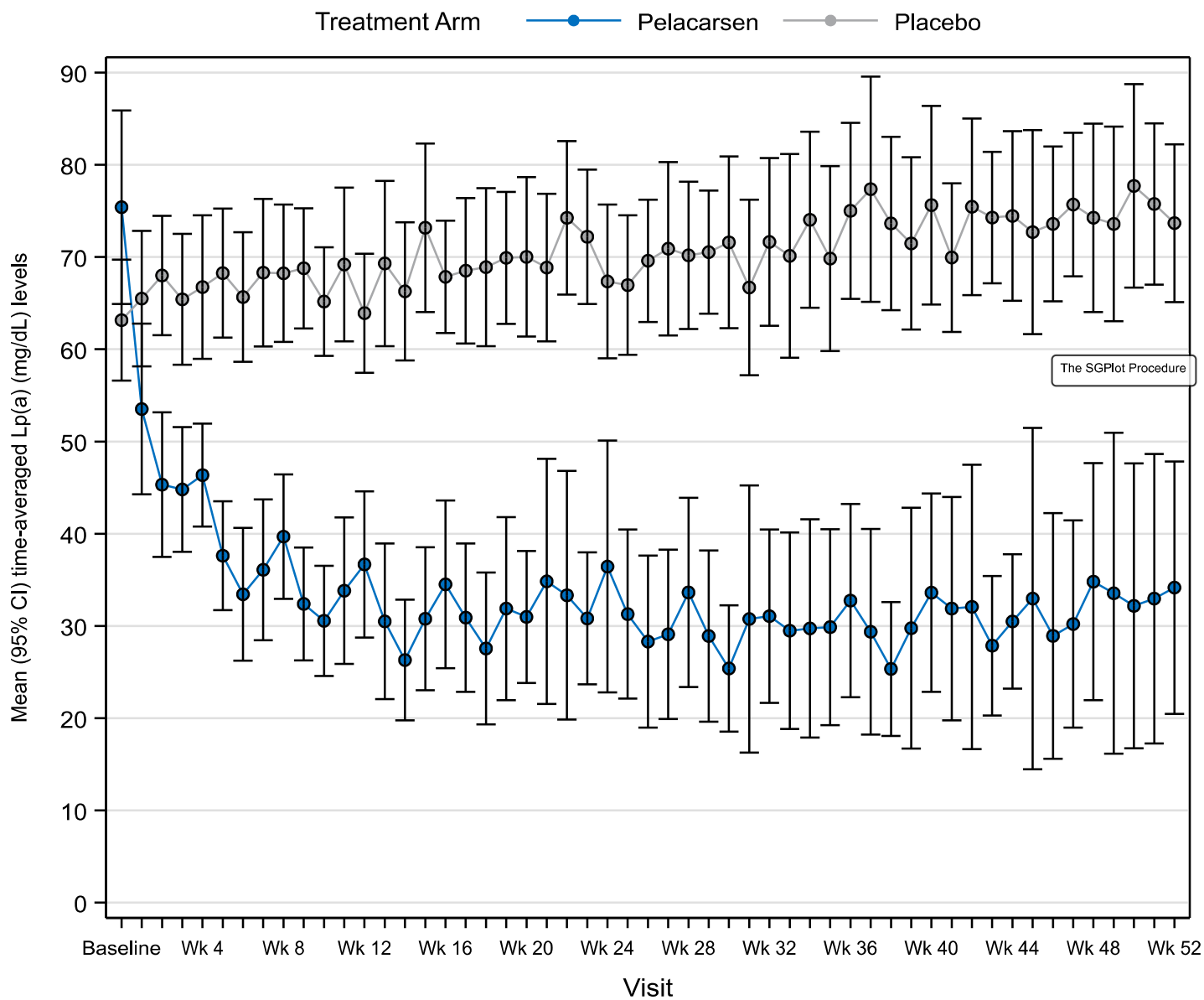

Supplement: ehag073_Supplementary_Data [file ehag073_supplementary_data.zip › Supplemental Figure 3.pdf]
